# A comparative study of the photosynthetic capacity in two green tide macroalgae using chlorophyll fluorescence

**DOI:** 10.1186/s40064-016-2488-7

**Published:** 2016-06-17

**Authors:** Ying Wang, Tongfei Qu, Xinyu Zhao, Xianghai Tang, Hui Xiao, Xuexi Tang

**Affiliations:** Department of Marine Ecology, College of Marine Life Sciences, Ocean University of China, Qingdao, 266003 China

**Keywords:** Yellow sea green tide, Chlorophyll fluorescence, Optimal photochemical efficiency, Quenching coefficient, 77 K chlorophyll fluorescence

## Abstract

Green tides have occurred in the Yellow Sea, China, every year from 2007 to 2015. The free-floating *Ulva prolifera* (Müller) J. Agardh was the causative macroalgal species. The co-occurring, attached *U. intestinalis* was also observed. Photosynthetic capacities were determined using chlorophyll fluorescence in situ and after 7 days lab acclimation, and a significant differences were noted. Pigment composition showed no obvious differences, but concentrations varied significantly, especially chlorophyll b in *U. prolifera* two times increase was observed after acclimation. The optimal photochemical efficiency of PS II (Fv/Fm) was significantly higher in *U. prolifera*. Photosynthetic rate (α), maximum relative electron transport rate (rETRmax), and minimum saturating irradiance (Ek), obtained from rapid light response curves (RLCs), showed almost the same photosynthetic physiological status as Fv/Fm. Quenching coefficients and low temperature (77 K) chlorophyll fluorescence emission spectra of thylakoid membranes analysis showed *U. prolifera* has a better recovery activity and plasticity of PSII than *U. intestinalis.* Furthermore, energy dissipation via non-photochemical quenching (NPQ) and state transitions showed efficacious photoprotection solution especially in *U. prolifera* suffered from the severe stresses. Results in the present study suggested that *U. prolifera*’s higher photosynthetic capacity would contribute to its free-floating proliferation, and efficacious photoprotection in addition to favorable oceanographic conditions and high nutrient levels support its growth and aggregation.

## Background

Green tides formed by some green algae excessive growth has been reported in in many parts of the world, including Europe, America, Australia, and Asia (Taylor et al. 2001; Nelson et al. 2003, 2008; Sun et al. 2008; Yabe et al. 2009; Kim et al. 2010; Zhang et al. 2013; Wang et al. 2012). In nutrient-rich habitats green algae grow rapidly and often cause marine fouling. Green tides have been the focus of many studies due to their detrimental effects on coastal ecology. Photosynthetic activities and capacities, proliferation, and nutrient absorption usually contribute to rapid biomass accumulation (Kim et al. 2010; Choi et al. 2010; Gao et al. 2010).

Large-scale green tides in the Yellow Sea of China, called “Yellow Sea green tides”, have occurred for 9 years from 2007 to 2015 (Sun et al. 2008; Liu et al. 2009; Wang et al. 2010, 2016; Luo and Liu 2011). The dominant species in these green tides was *Ulva prolifera* (Müller) J. Agardh (Sun et al. 2008; Ye et al. 2008; Leliaert et al. 2009). Phylogenetic analysis has suggested that this species is a unique strain within the *U. linza*-*procera*-*prolifera* (LPP) clade (Leliaert et al. 2009; Liu et al. 2010a). Another green tide alga in the area is *Ulva intestinalis*, a cosmopolitan species like *U. prolifera*, with mass occurrences recorded in the eutrophic estuaries of Europe and North America (Baeck et al. 2000; Cohen and Fong 2004). *U. intestinalis* always co-occurs with *U. prolifera*, but has not been recorded as a dominant species in the last four Yellow Sea green tides (Liu et al. 2010a). *U. prolifera* and *U. intestinalis* are benthic species, and usually aggregate by attaching to the bottom in intertidal zones, and forming colonies. However, during the Yellow Sea green tides of the last five years, *U. prolifera* has formed floating entangled colonies, while *U. intestinalis* retained attached to the bottom. However, little information is available to explain this relationship and the effects of the different free-floating and attached life-forms.

We suspected that the oxygen produced from photosynthesis would fill the inner tubes of *U. prolifera* and keep these buoyant. Then favorable oceanographic conditions and high nutrient levels in the Yellow Sea could support the floating *U. prolifera* to grow and aggregate and form a green tide. We thus deduced that there may be a close relationship between photosynthetic activities and capacities and the rapid accumulation of vast green algal biomass. The chlorophyll excitation energy dissipated by fluorescence has an inverse relationship with photosynthetic carbon assimilation (Schreiber 2004). Several selective measuring techniques have been developed to determine the photosynthetic rates, like PAM fluorometry and 77 K chlorophyll fluorescence. In recent years, many case studies of *Ulva* species (Chlorophyceae) have utilised these techniques for measuring the influences of environmental stress on PSII performance (Figueroa et al. 2003; Xia et al. 2004; Bischof et al. 2006; Choi et al. 2010; Luo and Liu 2011; Wang et al. 2012). Although there were differences between *Ulva* species, growth performance and PSII activity in the researched species were generally sensitive to stress conditions.

In this study we compared the photosynthetic performances of the free-floating *U. prolifera* and the attached *U. intestinalis* to investigate whether there was relationship between differing photosynthetic activities and differing life form. We mainly used chlorophyll fluorescence to indicate photosynthetic performance of thalli in the field, and after 7 days’ laboratory acclimation.

## Results

### Light absorption and chlorophyll content

The absorption spectra of the extracted pigments of *U. prolifera* and *U. intestinalis* exposed to different culture treatments were similar, which indicated that the pigment compositions of the samples were similar. Results of fluorescence scanning showed the maximum absorption spectra of chlorophyll a were 436 and 663 nm, while those of chlorophyll b were 463 and 645 nm.

There was significantly different chlorophyll content between the two species (two-way ANOVA, *p* < 0.0005), and the concentrations in *U. prolifera* were much higher than in *U. intestinalis* (Table 1, post hoc, *p* < 0.05). For chlorophyll a no significant differences were observed between the sampling phases (two-way ANOVA, *p* > 0.05) (Table 2), while the concentration of chlorophyll b was more affected by sampling phase than chlorophyll a (Table 2). Nevertheless, the difference between the chlorophyll a: b ratio in relation to environmental changes was not obvious.

**Table 1:** Mean concentration of photosynthetic pigments (n = 5) in *U. prolifera* and *U. intestinalis*, measured in situ and 7 days’ lab acclimation

Treatment	Chlorophyll a		Chlorophyll b		Chlorophyll	
Species	In-situ	After 7 days	In-situ	After 7 days	In-situ	After 7 days
*U. prolifera*	0.261^b^ ± 0.009	0.356^a^ ± 0.019	0.085^b^ ± 0.007	0.153^a^ ± 0.022	0.355^b^ ± 0.017	0.522^a^ ± 0.043
*U. intestinalis*	0.169 ^cd^ ± 0.043	0.115^d^ ± 0.013	0.051^c^ ± 0.017	0.044^c^ ± 0.018	0.226 ^cd^ ± 0.055	0.163^c^ ± 0.031

Values are means (± SD). Data are mg/fresh weight (g) (mg/g FW) for each pigment, different superscripts indicate significantly different values (*p* < 0.05) determined by post hoc comparisons

**Table 2:** Results of two-way ANOVA on effects of sampling phases (factor “phases”) and species (factor “species”) on the results of Chlorophyll a (Chl a), Chlorophyll b (Chl b), total Chlorophyll (Chl), Chlorophyll a: Chlorophyll b(Chla:Chlb), the optimal photochemical efficiency of photosystem II (Fv/Fm), maximum relative electron transport rate (rETRmax), photosynthetic rate in light-limited region of RLC (α) and minimum saturating irradiance Ek

Source	Dependent variable	*df*	F-ratio	*P* value
Phases	Chla	1	2.436	0.138
	Chlb	1	15.625	0.001
	Chl	1	8.844	0.009
	Chl a:b	1	4.854	0.043
	Fv/Fm	1	2.698	0.120
	rETRmax	1	42.057	0.000
	α	1	3.649	0.109
	Ek	1	11.375	0.007
Species	Chla	1	212.211	0.000
	Chlb	1	88.659	0.000
	Chl	1	197.113	0.000
	Chl a:b	1	2.863	0.110
	Fv/Fm	1	40.329	0.000
	rETRmax	1	80.594	0.000
	α	1	99.766	0.000
	Ek	1	19.429	0.000
Phases * species	Chla	1	38.977	0.000
	Chlb	1	24.252	0.000
	Chl	1	44.083	0.000
	Chl a:b	1	0.001	0.978
	Fv/Fm	1	0.065	0.801
	rETRmax	1	14.943	0.001
	α	1	5.437	0.035
	Ek	1	48.715	0.000
Error		16		

### Fluorescence descriptive parameters

#### Optimal photochemical efficiency

**Fig. 1:**
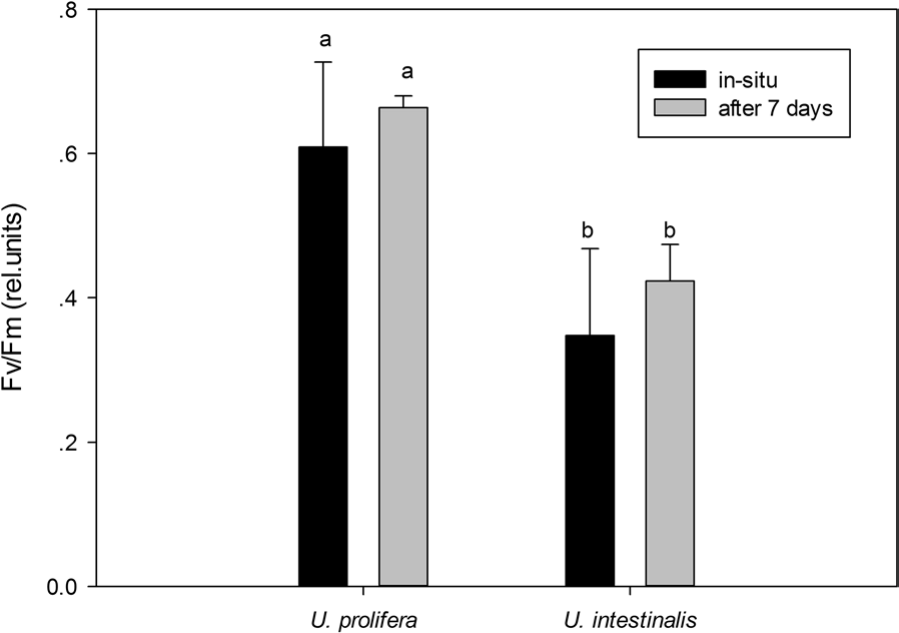
Mean optimal photochemical efficiency of photosynthesis (Fv/Fm) of *U. prolifera* and *U. intestinalis*, obtained from in situ and 7 days’ acclimation to each. Values are mean ± SD (n = 5). *Different letters* above *bars* indicate significantly different values (post hoc*, p* < 0.05)

Fv/Fm showed a pronounced difference between these two species (Table 2, two-way ANOVA, *p* < 0.0005). Fv/Fm of *U. prolifera* was significantly higher than that of *U. intestinalis*, both in situ and after acclimation (Fig. 1, post hoc, *p* < *0.05*). These two species had higher Fv/Fm values after acclimation, but these changes were not significant (Fig. 1, post hoc, *p* > 0.05).

#### Rapid light curves

**Fig. 2:**
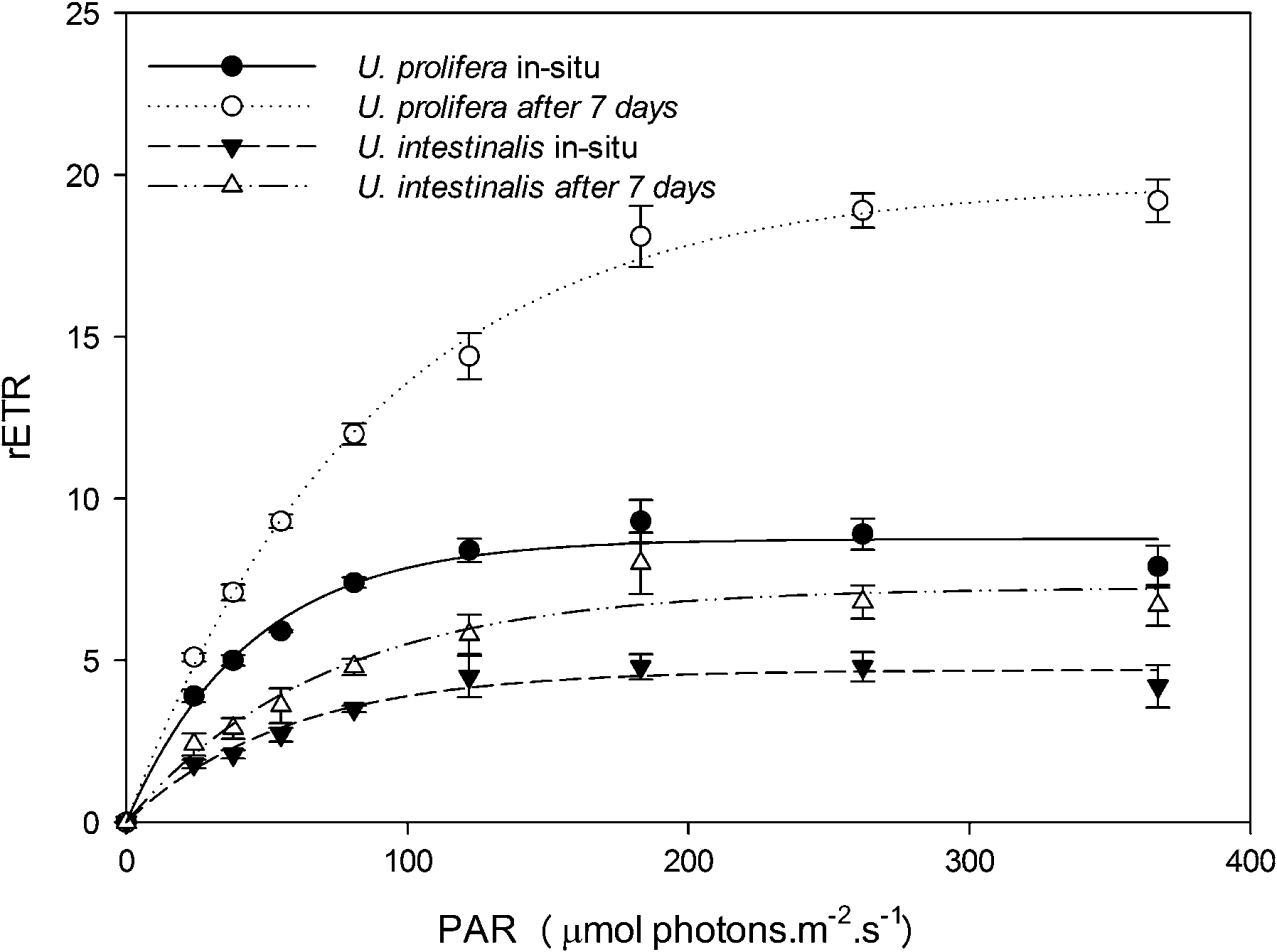
Mean relative electron transport rate (rETR) and fitted rapid light response curves (RLCs) of *U. prolifera* and *U. intestinalis*, obtained from in situ and 7 days’ acclimation to each. Values are mean ± SD (n = 5)

Figure 2 showed rETR with a linear rise until light was limiting, followed by a plateau where the photosynthetic pathway became limited. The mean rETRs of these two species increased and then slightly decreased at elevated irradiances, and the convexities of the curve were clearly greater in *U. prolifera* than in *U. intestinalis* (Fig. 2).

**Fig. 3:**
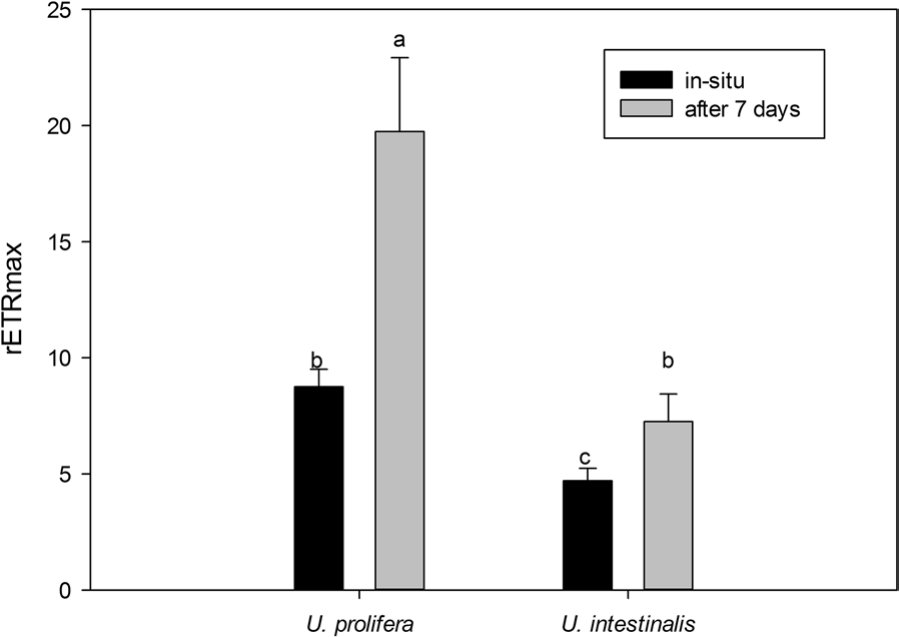
Mean maximum relative electron transport rate (rETRmax) obtained from fitted rapid light response curves (RLCs) of *U. prolifera* and *U. intestinalis*, measured in situ and 7 days’ acclimation to each. Values are mean ± SD (n = 5). *Different letters* above *bars* indicate significantly different values (post hoc*, p* < 0.05)

**Fig. 4:**
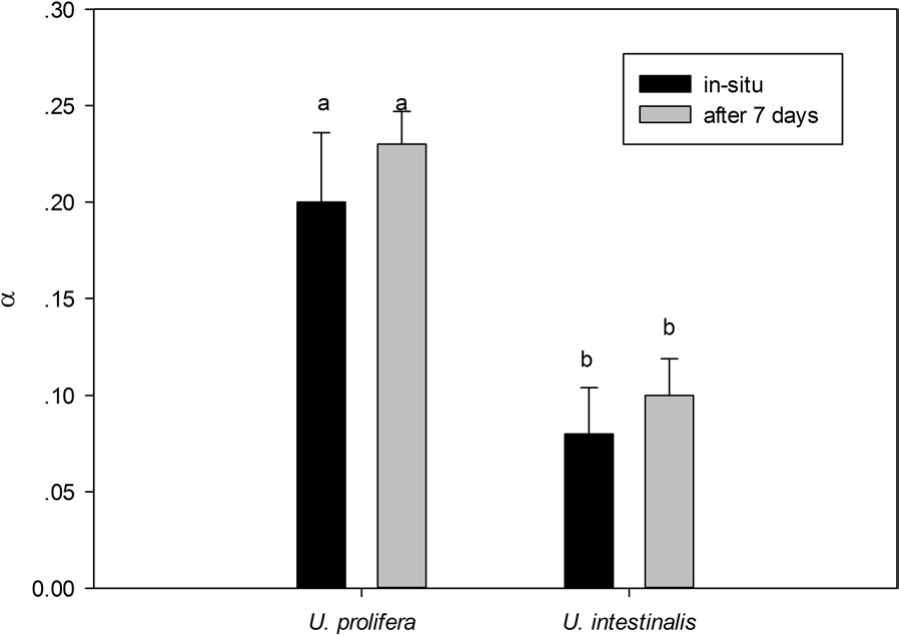
Mean photosynthetic rate in light-limited region of RLC (α) obtained from fitted rapid light response curves (RLCs) of *U. prolifera* and *U. intestinalis*, measured in situ and 7 days’ acclimation culture to each. Values are mean ± SD (n = 5). *Different letters* above *bars* indicate significantly different values (post hoc*, p* < 0.05)

**Fig. 5:**
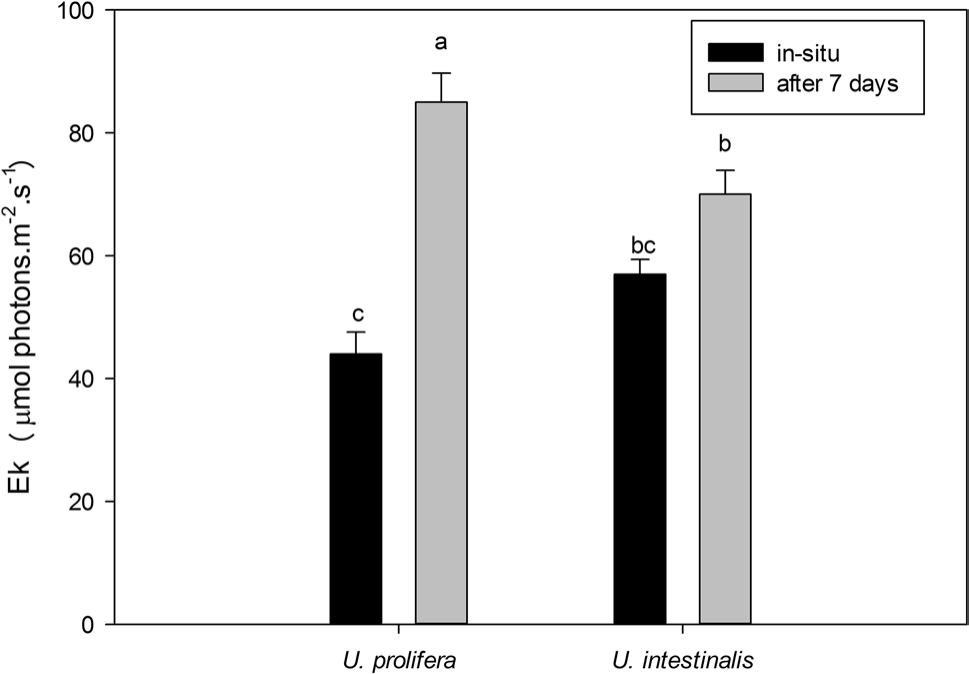
Mean minimum saturating irradiance Ek obtained from fitted rapid light response curves (RLCs) of *U. prolifera* and *U. intestinalis*, measured in situ and 7 days’ acclimation to each. Values are mean ± SD (n = 5). *Different letters* above *bars* indicate significantly different values (post hoc*, p* < 0.05)

From the fitted the curve we obtained the parameters α, rETRmax, and Ek. Then, we compared the exact photosynthetic performance by analyzing these parameters. These values in *U. prolifera* were significantly higher than those of *U. intestinalis* (Figs. 3, 4, 5), except for the parameter Ek in situ (Fig. 5, post hoc, *p* > 0.05). Moreover, the rETRmax and Ek of these two species were significantly greater in acclimated thalli than in those in situ (Figs. 3, 5).

#### Quenching coefficients

**Fig. 6:**
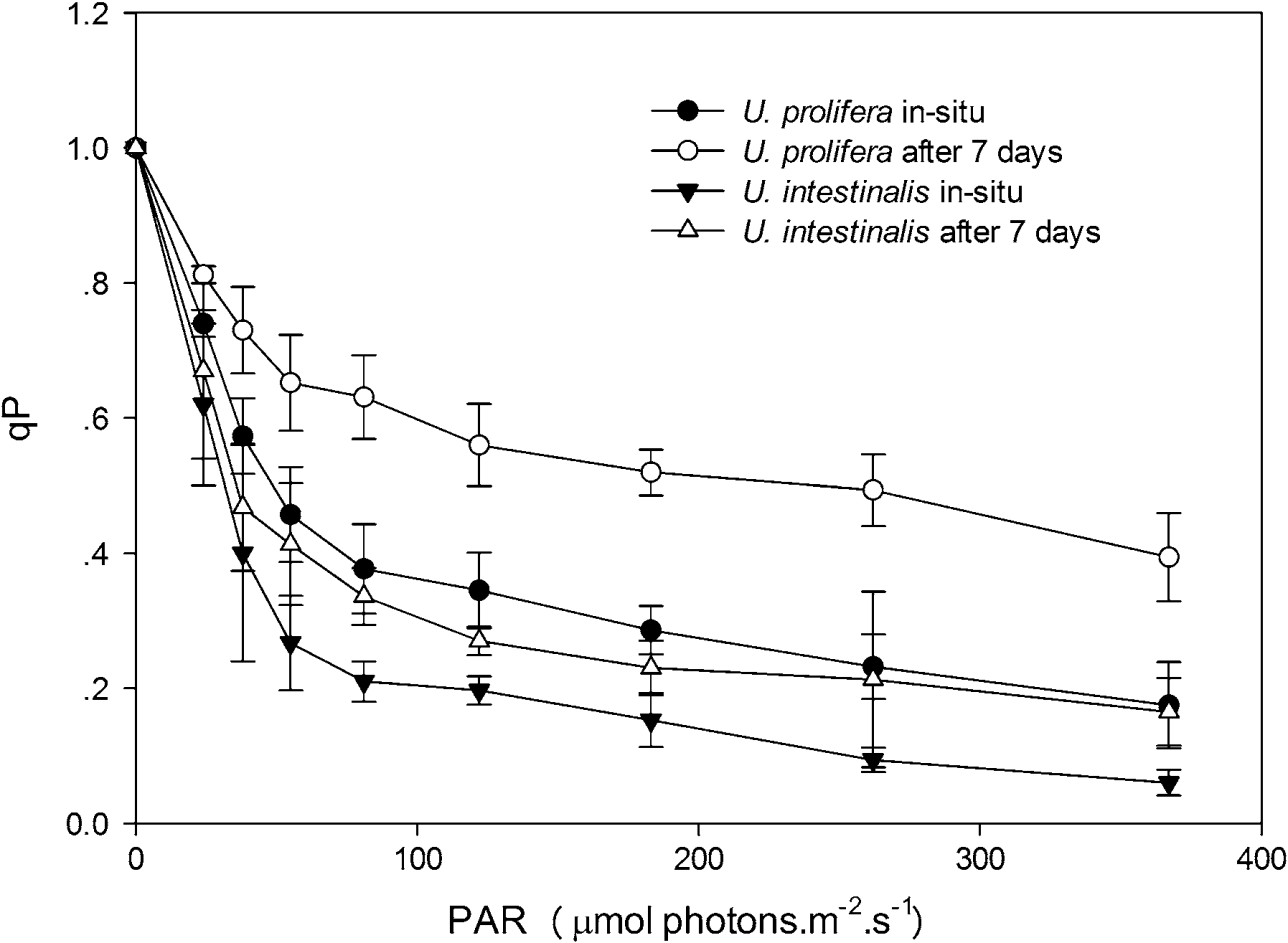
Quenching coefficient photochemical quenching (qP) as a function of increasing irradiances, obtained from in situ and 7 days’ acclimation culture to each. Values are mean ± SD (n = 5)

**Fig. 7:**
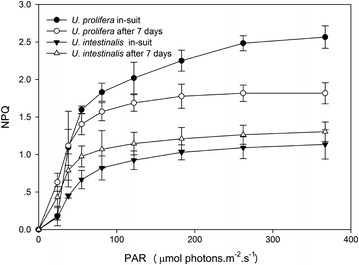
Quenching coefficient non-photochemical quenching (NPQ) a function of increasing irradiances, obtained from in situ and 7 days’ acclimation culture to each. Values are mean ± SD (n = 5)

Non-photochemical quenching (NPQ) and photochemical quenching (qP) extracted from recorded P-I curves indicated variations with respect to quantitative and qualitative changes in irradiance. NPQ showed similar patterns to the fitted RLCs of rETR. The NPQ and qP values of *U. prolifera* was higher than *U. intestinalis* at each light intensity (Figs. 6, 7).

## K Chlorophyll fluorescence emission spectra

**Fig. 8:**
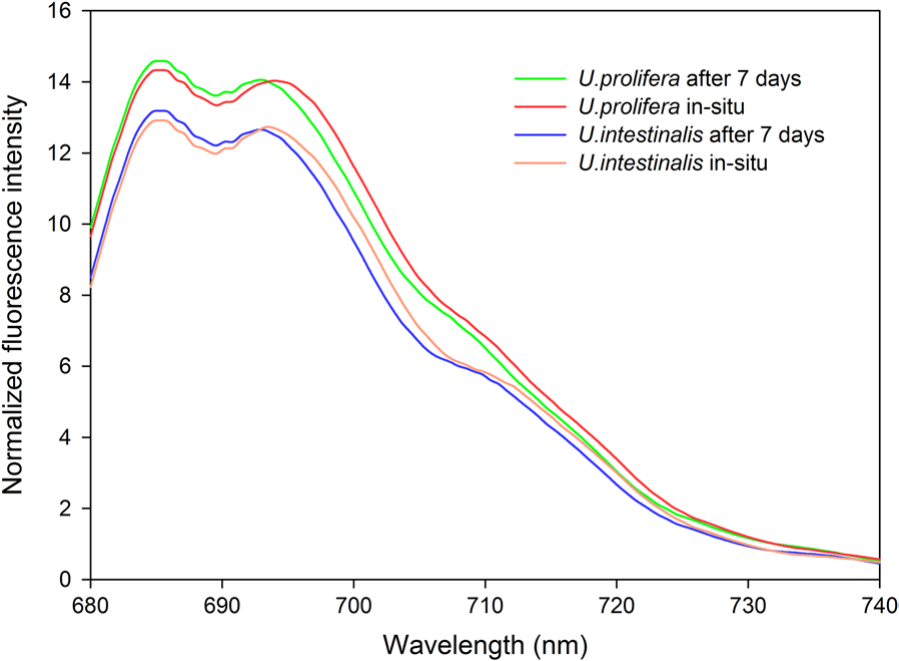
77 K chlorophyll fluorescence emission spectra of thylakoid membranes obtained from in situ and 7 days’ acclimation culture to each. Each curve was the mean of five independent experiments. The excitation wavelength was 436 nm

Figure 8 shows the changes in 77 K chlorophyll fluorescence emission spectra. The 77 K spectra had two dominant peaks at around 686 nm and 698 nm, and a shoulder peak at around 700–710 nm. The peaks of *U. prolifera* was higher than *U. intestinalis* both under in situ and after acclimation treatments (Fig. 8). A red shift of the major fluorescence peaks developed from lab acclimation (7 days treatment) to in situ status of these two macroalgae (Fig. 8).

## Discussion

Our study involved two phases: thallus measurements in situ and after 7 days lab acclimation. Some inhibiting effects in the field, such as nutrient limitation and herbivory, were eliminated after lab acclimation; the aim of acclimation was thus to gain potential and optimal performances. Pigment concentrations and chlorophyll fluorescence (rETRmax and Ek) of *U. prolifera* showed pronounced increases from in situ to lab acclimation (Table 1; Figs. 3, 5). *U. intestinalis* did not show these changes. This indicated that *U. prolifera* has a greater potential for achieving optimal photosynthetic performance. The reason for a slower response in *U. intestinalis* was probably insufficient time for acclimation, (pigment levels usually take several days to weeks to acclimate, as recognized in previous reports on *Ulva*) (Figueroa et al. 2003), or this species does not have as much photosynthetic plasticity as *U. prolifera*. The exact reason remains unknown. A similar result was shown by Luo and Liu (2011) for a salinity stress effect in *U. prolifera*, and by Bischof et al. (Bischof et al. 2006) for a UV-B stress effect in *U. lactuca*.

In this study a significantly higher chlorophyll concentration was found in *U. prolifera* than in *U. intestinalis* (Table 1). The same pattern is revealed by Fv/Fm, an indicator of photosynthetic physiological status. Chlorophyll level can directly determine potential photosynthesis (Lambers et al. 2008). Thus, the higher chlorophyll concentration partly contributed to higher photosynthesis in *U. prolifera*. We also found that an almost two times increase in *U. prolifera* chlorophyll b was observed after acclimation (Table 2). Chorophyll b as accessory pigments usually serve to increase the efficiency of photosynthesis by enhancing PS II conversion of light to chemical energy, hence this is a reason for a potentially higher photosynthetic rate in *U. prolifera*. Furthermore, another result rETR increases in both species following incubation in the lab under a much lower photon flux than the natural environment. Perhaps Chorophyll b contributed light acclimation of the algae in this process. In addition to the chlorophyll concentration discussed above, another factor that may explain the difference in photosynthetic efficiency is thallus morphology. Previous studies (Johansson and Snoeijs 2002; Rautenberger et al. 2009) have found that photosynthetic capacity depends on thallus morphology: thinner, more filamentous species were found to have higher photosynthetic rates than coarser, thicker species. Therefore, difference between filamentous *U. prolifera* and tubular *U. intestinalis* is an important influence on photosynthetic capacity.

The results of the chlorophyll fluorescence investigation could further demonstrate that *U. prolifera* had a more flexible photosynthetic capacity than *U. intestinalis*. Fv/Fm is a parameter that can reflect the maximum quantum yield of PSII. Under the normal condition the mean value of Fv/Fm in green plants is around 0.7 (Kalaji et al. 2014). In this study, Fv/Fm in *U. prolifera* maintained around 0.7 suggesting that *U. prolifera* had a stable photosynthetic efficiency (Fig. 1). But the mean Fv/Fm of *U. intestinalis* under in situ and after 7 day acclimation was 0.4 which was very low value compared with other green algae. This indicated that the *U. intestinalis* was highly stressed and simply did not recover rapidly enough within the 7 day lab acclimation. The fitted RLCs. curves showed that *U. prolifera* showed a steady and sharper increase in rETR in comparison with *U. intestinalis* (Fig. 2). The more rapid changes in rETR in *U.prolifera* may point to a more plastic response than in *U. intestinalis*. Therefore, stable photosynthetic capacity in *U. prolifera* causes it to produce more oxygen and which fills its inner tubes and keeps them buoyant. And flexible photosynthetic plasticity in *U. prolifera* causes it cope with the changing environmental stress on the sea surface.

We found that the quenching coefficients (qP and NPQ) of these two *Ulva* species were related to the rETR. The RLCs curves showed a slight down-turn at elevated irradiances (Fig. 2). Lavaud et al. suggested that in RLCs this decline may be better linked to dynamic down-regulation of Photosystem II (Lavaud et al. 2004). A decline in rETR is generally attributable to increased energy dissipation via non-photochemical quenching (NPQ) (Ralph et al. 2002). In this study *U. prolifera* showed a higher increase in NPQ and a lower decrease in qP with increasing irradiance than *U. intestinalis* (Figs. 6, 7). This may suggest more efficacious photoprotection in the former than in the latter. The free-floating *U. prolifera* suffers more complex and harsh environmental conditions including higher irradiance than the attached *U. intestinalis*, so more efficacious photoprotection is very important to *U. prolifera*, and further helps explain its green tide forming potential.

In this study, we also analyzed 77 K chlorophyll fluorescence emission spectra of thylakoid membrane of these two macroalgae. In this study the 77 K emission spectra peaks were at around 686 and 698 nm (Fig. 8), which emitted mainly from CP43 and CP47 in PSII core complexes. Unlike other terrestrial plants there is no dominant peak at around 730 nm which is related to the antenna of PSI, but a shoulder peak at around 700–710 nm can be observed (Fig. 8). Previous study showed the shoulder peak was related to aggregate LHCII (Kalaji et al. 2014). Thus, the 77 K chlorophyll fluorescence emission spectra peaks of these two macroalgae are due to PSII (CP43, CP47 and LHCII). Figure 8 showed the peaks of *U. prolifera* was higher than *U. intestinalis* both under in situ and after acclimation treatments, which suggests *U. prolifera* has a better recovery activity and plasticity of PSII than *U.intestinalis.*

Another common application of 77 K chlorophyll fluorescence measurements is to detect the occurrence of state transitions (Bellafiore et al. 2005; Drop et al. 2014). In this study a red shift of the major fluorescence peaks developed from lab acclimation (7 days treatment) to in situ status of these two macroalgae (Fig. 8), which suggests that state transitions occurred. State transitions represent a photoprotection process that regulates the light-driven photosynthetic reactions in response to changes in light quality/quantity (Drop et al. 2014). The most likely explanation of the red shift is that thallus in situ migrated excitation energy towards long wavelength absorbing represents. So state transitions showed another efficacious photoprotection solution especially in *U. prolifera* suffered from the most severe stresses, such as high light, high temperature. The displacement of LHCII or PSI may contribute to this red shift, but the exact explanation for the state transition in this study need further investigated.

## Conclusions

Compared with the co-occurring *U. intestinalis*, higher photosynthetic capacity in *U. prolifera* contributes to its free-floating lifestyle during Yellow Sea green tides, which prevents *U. prolifera* from sinking out of the euphotic zone. Furthermore, more efficacious photoprotection via state transition and non-photochemical quenching is able to support the proliferation and aggregation of *U. prolifera* to form green tides.

## Methods

### Sampling and culture conditions

The thalli of *U. prolifera* and *U. intestinalis* were collected from coastal Qingdao (36.0492’N, 120.3536’E) in June, 2010, during a bloom period.

Thalli were gently rinsed in sterile seawater and thoroughly cleaned with a brush under a magnifier to remove attached sediment, small grazers, and epiphytes. Thalli were lab acclimated by culturing in sterile seawater enriched with f/2 medium (Guillard 1975), at a constant 20 °C and a light intensity of 72 μmol photons m^−2^ s^−1^, in a 12:12 h light:dark cycle using a GXZ-280C intelligent illumination incubator (Ningbo Jiangnan Instrument, China). Germanium dioxide (GeO_2_) at a concentration of 0.5 mg l^−1^ was added to the cultures to suppress diatom growth (Lotze et al. 2000). The culture medium was completely renewed every two days.

### Experimental design

This experimental had two sampling phases: in situ and after 7 days lab acclimated. In situ sampling was to compare the photosynthetic capacity of the two species under the natural condition; after 7 days lab acclimation was to determine the recovery capacity differences of the two species. Five healthy thalli groups of each species were randomly selected as experimental groups during the green tide. Firstly, thalli from the experimental groups were treated in situ to determine pigments and chlorophyll fluorescence, and then transferred the experimental groups to the lab for acclimation. Lab acclimation lasted for 7 days. After acclimation thalli from the experimental groups were treated again to re-determine pigments and chlorophyll fluorescence.

### Light absorption analysis and chlorophyll content determination

0.5 g fresh weight (FW) thallus samples were ground in liquid nitrogen and extracted using 90 % (V/V) acetone buffer (5 ml). The acquired mixture was then subjected to 6000 g at room temperature for 10 min and the supernatant was used for further analyses. Light absorption and chlorophyll content were determined using a Hitachi F-4500 Fluorescence Spectrophotometer (HITACHI, Japan) and scanning absorption spectra of 350–700 nm. Levels were calculated using the following formulae (Arnon 1949):

1 $${\text{Chl a }} = { 12}. 7 {\text{A}}_{ 6 6 3} - 2. 6 9 {\text{A}}_{ 6 4 5}$$

2 $${\text{Chl b }} = { 22}. 9 {\text{A}}_{ 6 4 5} - 4. 6 8 {\text{A}}_{ 6 6 3}$$

3 $${\text{Chl }} = { 8}.0 2 {\text{A}}_{ 6 6 3} + { 21}. 2 1 {\text{A}}_{ 6 4 5}$$

All assays were performed in triplicate. Results are expressed as milligrams per gram of fresh weight (mg/g FW).

### Thylakoid membrane preparation

1 g fresh weight (FW) thallus samples were ground in liquid nitrogen and then homogenized in a medium (pH 7.6) containing 50 mM Tricine, 0.4 M sucrose. The thylakoid membrane was prepared as described previously (Tang et al. 2005) with minor modifications and carried under 0–4 °C. The three centrifugations were modified as 500*g* for 5 min, 8000*g* for 10 min, 13,000*g* for 20 min. Prepared thylakoid membrane were stored in 10 % glycerol at −80 °C for further use.

### Chlorophyll fluorescence measurements

In vivo chlorophyll fluorescence analyses included measurement of optimal photochemical efficiency (Fv/Fm), creation of rapid light response curves (RLCs), quenching coefficients, and 77 K chlorophyll fluorescence emission spectra measurements.

Photosynthetic efficiency was determined using a portable pulse amplitude modulated (PAM) fluorometer (Mini PAM, Walz, Germany). The general protocol we used to determine chlorophyll fluorescence followed Wang et al. (2012) and the Mini-PAM operational handbook. Samples were determined in 5 replicates, and measurements were not repeated on the same tissue during a time course. The Fv/Fm can be obtained from the equation: ${\text{F}}_{\text{v}} /{\text{F}}_{\text{m}} = \left( {{\text{F}}_{\text{m}} - {\text{F}}_{0} } \right)/{\text{Fm}}$. In this equation, F_0_ is the minimal fluorescence after dark acclimation and F_m_ means the maximal fluorescence after saturation flashes in the dark-acclimatized sample.

RLCs were subsequently measured. Samples were exposed to a light intensity gradient (PAR 0, 24, 38, 55, 81, 122, 183, 262 and 367 μmol photons m^−2^ s^−1^), with each step lasting for 10 s. Simultaneously, the relative electron transport rate (rETR) and the effective photosynthetic yield (Y(II)) of photosystem II (PSII) were measured under each light intensity. RLCs were fitted using the equation of (Platt et al. 1980) to determine photosynthetic rate in the light-limited regions of RLCs (α), maximum relative electron transport rate (rETRmax), and minimum saturating irradiance (Ek). The regression algorithm was:

4 $${\text{P}} = {\text{P}}_{\text{m}} \times \left( { 1- {\text{e}}^{{ - \alpha {\text{Ed}}/{\text{Ps}}}} } \right)$$

where Pm the photosynthetic capacity at saturating light, α initial slope of the RLC before the onset of saturation. rETRmax and Ek were estimated using the following equations:

5 $${\text{rETRmax}} = {\text{P}}_{\text{m}} \times (\alpha /[\alpha + \beta ]) \times (\beta /[\alpha + \beta ])^{\beta /\alpha }$$

6 $$\text{Ek} = \text{ETRmax} /{\upalpha }$$

Photochemical (qP) and non-photochemical (NPQ) quenching coefficients were calculated based on a built-in function of the Mini-PAM and the following equations:

7 $${\text{qP}} = \left( {{\text{Fm}}^{\prime } - {\text{Ft}}} \right)/\left( {{\text{Fm}}^{\prime } - {\text{F}}0} \right)$$

8 $$\text{NPQ} = (\text{Fm} - \text{Fm}^{\prime})/\text{Fm}^{\prime}$$

77 K chlorophyll fluorescence emission spectra of thylakoid membrane were recorded with a Hitachi F-4500 Fluorescence Spectrophotometer (HITACHI, Japan). The excitation wavelength was 436 nm (slit 5 nm) and the emission was recorded between 680 and 740 nm (slit 1.2 nm).

### Statistic analysis

The values of chlorophyll levels and fluorescence descriptive parameters were statistically compared using a two-way ANOVA. The Student–Newman–Keuls post hoc multiple comparison test and Duncan’s post hoc test were used if ANOVA indicated a significant effect. Differences between treatment means are considered significant if *p* < 0.05. Data were analyzed using IBM SPSS Statistics 19 (SPSS Inc, USA). All values cited in this paper were obtained from fully independent samples.
